# Will the EU Medical Device Regulation help to improve the safety and performance of medical AI devices?

**DOI:** 10.1177/20552076221089079

**Published:** 2022-03-30

**Authors:** Emilia Niemiec

**Affiliations:** Medical Ethics Division, Department of Clinical Sciences, 5193 Lund University, Sweden

**Keywords:** Medical device regulation, artificial intelligence

## Abstract

Concerns have been raised over the quality of evidence on the performance of medical artificial intelligence devices, including devices that are already on the market in the USA and Europe. Recently, the Medical Device Regulation, which aims to set high standards of safety and quality, has become applicable in the European Union. The aim of this article is to discuss whether, and how, the Medical Device Regulation will help improve the safety and performance of medical artificial intelligence devices entering the market. The Medical Device Regulation introduces new rules for risk classification of the devices, which will result in more devices subjected to a higher degree of scrutiny before entering the market; more stringent requirements on clinical evaluation, including the requirement for appraisal of clinical data; new requirements for post-market surveillance, which may help spot early on any new, unexpected side effects and risks of the devices; and requirements for notified bodies, including for expertise of the personnel and consideration of relevant best practice documents. The guidance of the Medical Device Coordination Group on clinical evaluation of medical device software and the MEDDEV2.7 guideline on clinical evaluation also attend to some of the problems identified in studies on medical artificial intelligence devices. The Medical Device Regulation will likely help improve the safety and performance of the medical artificial intelligence devices on the European market. The impact of the Regulation, however, is also dependent on its adequate enforcement by the European Union member states.

Artificial intelligence (AI)-based technologies are being developed to improve various areas of medical care, including diagnostics, surgery and healthcare system management.^1,2^ Although recent improvements in these technologies have been significant and the number of devices on the market has grown,^3^ concerns have been raised over the quality of evidence on the performance of medical AI.^4–9^ Problems have been revealed both with AI solutions that are possibly still under development^4–7^ and those already available on the market in the USA and Europe.^8,9^ The issues identified include lack of external validation of the systems (with the same dataset being used for training and validation),^5,7^ which may lead to overestimates of diagnostic accuracy; limited numbers of randomised clinical trials^5^ and multi-site evaluations^8,9^; a preponderance of retrospective studies (i.e. using previously collected datasets) over prospective studies run in a real clinical environment^5,7–9^; heterogeneity in the metrics used, which makes the comparison of systems difficult^6,7^; and lack of availability of the datasets and codes that would permit studies to be replicated.^5^ Owing to these shortcomings, the performance and safety claimed by the developers of the tested AI systems may be overestimated. This may, in turn, translate into the risk of harm to patients, and wasteful use of healthcare resources, if the devices are used in medical practice.

In response to these issues, the academic community has been working on standards for the various types of studies in which AI systems are developed and tested.^10–16^ Educational articles and guidelines for healthcare professionals have also been published to aid decision-making on the use of AI tools.^17–21^

The question of standards for medical AI devices^*^ has also been of interest to regulators. In January 2021, the US Food and Drug Administration (FDA) issued the ‘Artificial Intelligence/Machine Learning (AI/ML)-Based Software as a Medical Device (SaMD) Action Plan’. In this document, the FDA declares support for, among other things, efforts to develop ‘methodology for the evaluation and improvement of machine learning algorithms, including for the identification and elimination of bias, and on the robustness and resilience of these algorithms to withstand changing clinical inputs and conditions’.^22^ Meanwhile, in Europe, a new Medical Device Regulation (MDR) applying to medical AI devices became applicable on 26 May 2021.^23,24^ A few authors have examined the impact of the MDR on medical AI devices, and the requirements that have to be met under this Regulation.^25–27^ To date no published article, however, has specifically discussed whether, and how, the MDR will improve the safety and performance of medical AI devices entering the market. The aim of this article is to address this question. After providing a brief overview of the MDR, the article focuses on the changes introduced by the MDR that seem most relevant to improving the safety and performance of medical AI devices. These include changes in risk classification (and consequently the applicable conformity assessment procedures), clinical evaluation, post-market surveillance and notified bodies (NBs). These issues (or some of their aspects) are underlined in the MDR preamble as the ‘key elements’ of the regulatory system that should be reinforced ‘to improve health and safety’ (Recital 4 of the MDR). The impact of changes in risk classification, clinical evaluation and post-market surveillance on medical AI devices was also highlighted by other authors.^26,27^

## The MDR – an overview

The MDR entered into force in May 2017, replacing the Medical Device Directive (MDD) and the Active Implantable Medical Device Directive. The MDR applies from 26 May 2021, with the exception of some provisions (Article 123).^23,24^ The new Regulation is a response to technological progress in the development of medical devices. It also addresses problems with the previous regulatory system revealed in scandals involving unsafe medical devices, and it harmonises the rules for medical devices in the EU.^28^ Unlike directives, regulations apply directly in the EU member states, that is, without being implemented in national law. This means that after the four-year transition period, from 26 May 2021, new medical devices must comply with the MDR if they are to be marketed and sold. However, certificates of conformity (required to place a medical device on the market) issued earlier, under the MDD, will remain valid as indicated on the certificate, but no longer than until 27 May 2022 or 24 May 2024 (depending on the type of the certificate, Article 120 of the MDR).

Core elements of the MDD are maintained in the MDR. Under both, medical devices must meet a set of requirements to enter the market. In the MDD these are termed ‘essential requirements’, while in the MDR they are referred to as ‘general safety and performance requirements’. Both documents require devices to achieve the performance claimed by the manufacturer, and that the safety risks are acceptable in relation to benefits. Compliance with the requirements is verified in a conformity assessment procedure. Both MDD and MDR categorise medical devices into risk classes. The conformity of devices with the lowest risks can be self-declared by the manufacturer. In the case of higher-risk devices, compliance with the requirements needs to be verified by a notified body – that is, a private company designated to conduct conformity assessments. Conformity assessment procedures in the MDR are similar to those in the MDD. Manufacturers of higher-risk devices can choose among different conformity assessment procedures, depending on the risk class of the device. Devices passing the conformity assessment receive a CE (Conformité Européenne) mark and can be placed on the market or put into service.

Although the main principles of MDD and MDR are similar, there are important differences in the requirements set by the documents.

## Risk classes

Many devices that under the MDD fell into the lowest risk class will now shift to a higher risk class.^26^ The MDR addresses risk classification of software in a separate rule (Rule 11), although other classification rules may also apply (Rules 9, 10, 12 and 13).^27^ This risk categorisation for software is in line with the International Medical Device Regulators Forum approach, which factors in ‘A. [s]ignificance of the information provided by the SaMD [Software as a Medical Device] to the healthcare decision, and B. [s]tate of the healthcare situation or condition’.^29^ Both these factors depend on the intended use defined by the manufacturer and not on the technology used. Rule 11 in the MDR states that:

Software intended to provide information which is used to take decisions with diagnosis or therapeutic purposes is classified as class IIa, except if such decisions have an impact that may cause*:*

- death or an irreversible deterioration of a person's state of health, in which case it is in class III; or

- a serious deterioration of a person's state of health or a surgical intervention, in which case it is classified as class IIb.

Software intended to monitor physiological processes is classified as class IIa, except if it is intended for monitoring of vital physiological parameters, where the nature of variations of those parameters is such that it could result in immediate danger to the patient, in which case it is classified as class IIb.

All other software is classified as class I. (MDR Annex VIII, section 6.3, Rule 11)

Based on this rule, most AI devices will probably be classified as Class IIa or IIb,^26^ and unlike Class I devices they will therefore be required to undergo conformity assessment by a notified body. In other words, under the MDR more devices will be subjected to a higher degree of scrutiny before entering the market. This, in consequence, will ideally help to ensure that devices entering the market demonstrate the performance and safety claimed by the manufacturer.

## Clinical evaluation

In the MDR, clinical evaluation is required regardless of the risk class of the device. The goal of this evaluation is to verify compliance with the general safety and performance requirements (Article 61). The requirements include, among other things, the ability of the device to deliver its intended performance, its safety, and the acceptability of the benefit–risk ratio in the context of the state-of-the-art (Annex I). In comparison with the MDD, the MDR raises the level of the requirements to be met in clinical evaluation and places more emphasis on clinical evidence collected after the device has been placed on the market.

The Regulation provides more detailed requirements for clinical evaluation than those imposed by the MDD. It does so in part by incorporating elements of a non-legally binding guideline on clinical evaluation issued in 2016 under the MDD (MEDDEV 2.7/1 (rev. 4)).^30^ MEDDEV 2.7/1 (rev. 4) is the most recent guideline on clinical evaluation and it is in keeping with the requirements of the MDR. It explains general principles of clinical evaluation and describes each stage of the clinical evaluation process. The steps of clinical evaluation for medical devices outlined in the MDR and described in more detail in the MEDDEV 2.7 include (1) preparation (and updating) of a clinical evaluation plan; (2) identification of relevant clinical data (both generated by the manufacturer and available in scientific literature) and of gaps in the available evidence through a systematic literature review; (3) appraisal of the available data, including the methodological quality and scientific validity, as well as their relevance to the clinical evaluation of the device; (4) clinical investigation (involving human subjects), which is mandatory for class III and implantable devices, but necessary for other devices only if it is needed to address gaps in the evidence; (5) analysis of all relevant clinical data to evaluate whether they demonstrate conformity with the general safety and performance requirements (Annex XIV of the MDR).^30^ Both the MDD and the MDR allow manufacturers to claim equivalence of their device under evaluation to a device that is already CE-marked and use clinical data of that CE-marked device for clinical evaluation. The requirements of the MDR (Annex XIV) are, however, higher than in the MDD, and it seems unlikely that manufacturers of medical AI devices will use this option.^31^ Results of the clinical evaluation should be presented in a clinical evaluation report, which is part of the technical documentation required for conformity assessment (together with other elements that depend on the risk class of the device) (Annex II, XIV). The requirements for clinical evaluation in the Regulation, in particular the requirement for the appraisal of clinical data, may increase the attention paid by manufacturers and NBs to the quality of clinical evaluation and contribute to its improvement.

MEDDEV 2.7/1 (rev. 4), in section ‘9. Appraisal of pertinent data (Stage 2)’ offers further guidance on the evaluation of the methodological quality of studies. It mentions that the possibility of confounding influences, bias, random error, lack of transparency in reporting and misinterpretation should be considered in the evaluation. It also outlines specific issues that should be critically appraised, such as the appropriateness of sample size, the power of calculations, the endpoints and controls used, and the validity of conclusions.

There is also another guidance document on clinical evaluation, which specifically addresses the clinical evaluation of software qualifying as a medical device (i.e. so-called medical device software).^32^ It was developed by the Medical Device Coordination Group (MDCG) established by Article 103 of the MDR. The guidance also applies to medical AI devices. The drafting here draws on the approach developed by the International Medical Device Regulators Forum^33^ and outlines three components of clinical evidence for medical device software: valid clinical association, technical performance and clinical performance. Data supporting these three types of clinical evidence need to be identified or generated, to show compliance with the applicable general safety and performance requirements. The document provides general recommendations on the type and quality of the studies that may be involved here, as well as measurements that can be used to demonstrate valid clinical association, technical performance and clinical performance.

Where the quality of the studies is concerned, the MDCG guidance proposes questions that can be used in their evaluation. For example:

Were the type and the design of the study/ test appropriate to meet the research objectives? Was the data set appropriate and actual (state of the art)? Was the statistical approach appropriate to reach a valid conclusion?^32^

Attention to these aspects may indeed help users to conduct methodologically sound studies. On the other hand, these are rather general recommendations.

Importantly, the document also explains when prospective studies may be required and stresses that data for retrospective analyses should be of appropriate quantity and quality:

If the MDSW [medical device software] is used for the determination of a patient's future state (e.g. predisposition, prognosis, prediction) or if the output of the MDSW impacts clinical outcomes (e.g. treatment efficacy) or patient management decisions, then a prospective study may be required as part of the device's clinical evaluation (MDR)/performance evaluation (IVDR [In Vitro Diagnostic Medical Devices Regulation]). In other situations, retrospective analysis may be more appropriate to generate the necessary data to support compliance with the GSPRs [general safety and performance requirements], as there is no impact on patient management and the research does not introduce any risks to the patients. Such an approach is only possible under condition that there is an adequate access to data sets of sufficient amount and quality and obtained from the target population*.*^32^

The document then presents a few examples to illustrate different strategies for the clinical evaluation, including the use of prospective and retrospective studies. These recommendations may help address the lack of prospective studies on medical AI. However, one could argue that in the context of medical AI devices, a stronger stance in favour of the need for prospective studies would be needed. Retrospective studies might not adequately reflect real-world clinical settings and consequently might overestimate the performance of the device.^5,34^ Therefore, as claimed by Topol, ‘[p]rospective trials that are representative of patient care are essential’, while ‘[r]etrospective studies of AI in healthcare must … be considered hypothesis generating, often a best-case scenario, and unacceptable as definitive proof points’.^34^ The MDCG's guidance also does not specify further what kind of prospective studies should be conducted (e.g. studies in real clinical settings, randomized clinical trials and multi-site studies) and when. The document, however, does not focus on AI devices and is not legally binding; it is, therefore, understandable that it was formulated to allow for flexibility.

Although the guidance document states that retrospective studies may be sufficient in some cases, it also outlines that the usability assessment is part of clinical performance evaluation. Indeed, in the MDR, requirements on usability are a subset of the general safety and performance requirements. They, in part, overlap with the requirements of the MDD, but in the MDR they are more extensive. Manufacturers should, among other things, assess risks related to the intended use as well as potential misuse of the device and minimise the risks related to ergonomic features of the device and the environment in which it is supposed to be used (Annex I). Characteristics of users of the device, for example, their technical knowledge and training, should also be accounted for in the design. To demonstrate compliance with these requirements manufacturers can implement international standard IEC 62366: ‘Applications of usability engineering to medical devices’.^38^ The standard outlines two types of evaluation of interactions by users with the user interface: formative and summative. Formative evaluation is conducted iteratively at the stage of the device's design and development, while summative evaluation should be performed once the user interface is developed to test hazard-related use scenarios and obtain objective evidence of the safety of the user interface. Summative evaluation should be conducted ‘in a simulated or actual user environment’ (Annex II). Such an evaluation provides insight into the interaction of a clinician (or another intended user) with the device and, in the context of medical AI devices, in some respects may compensate for a lack of prospective studies in real clinical settings.

To recapitulate the MEDDEV 2.7 guideline and the MDCG's guidance, both emphasise issues related to the quality of research on the performance and safety relevant to medical AI. They directly address several weaknesses of studies that have been identified by the academic community. Drawing the attention of both manufacturers and notified bodies to these issues may help address them. However, the documents do not specifically address some problems connected to AI research, such as the lack of an adequate, external validation of the systems deploying a dataset other than the one used for training, as well as the lack of randomised clinical trials and multi-site evaluations. Rather, they provide general recommendations. Furthermore, they are not legally binding, and the extent of their implementation is uncertain.

## Post-market surveillance

Post-market surveillance is mentioned in the MDD. However, it is not defined there, and the Directive offers limited guidance on it (Annex II, Annex X). The MDR, by contrast, introduces more detailed and stringent rules on post-market surveillance.

The MDR requires manufacturers to establish a post-market surveillance system that should ‘be suited to actively and systematically gathering, recording and analysing relevant data on the quality, performance and safety of a device throughout its entire lifetime, and to drawing the necessary conclusions and to determining, implementing and monitoring any preventive and corrective actions’ (Article 83(2)). In particular, the data should be used to update the benefit-risk determination, the clinical evaluation, the summary of safety and clinical performance, and other matters (Article 83 (3)).

The post-market surveillance plan should be included as part of the technical documentation required for conformity assessment. The plan should address, for example, how the relevant data will be collected and evaluated, information about the benefit–risk reassessment, methods for the investigation of complaints and management of incidents, and the post-market clinical follow-up plan (Annex III). The post-market clinical follow-up plan, in turn, should, among other things, describe how clinical data will be collected and evaluated throughout the lifetime of the device, how new side-effects, emergent risks and off-label use of the device will be identified, and how the acceptability of the benefit–risk ratio will be ensured (Annex XVI, 6.1).

The manufacturers should prepare a report containing a summary of the results and the conclusions of post-market analysis together with ‘a rationale and description of any preventive and corrective actions taken’ (Articles 85 and 86)*.* The report should be updated with a frequency that depends on the class of the device (Articles 85 and 86).

To facilitate the traceability of devices and post-market surveillance, the MDR introduced a Unique Device Identification system (UDI system) within which each device receives a unique identifier (Article 27).

The post-market surveillance system required by the MDR should help manufacturers of medical AI devices to spot early on any new, unexpected side effects and risks of the devices and take corrective actions, which may improve the overall safety of devices on the market.

## Notified bodies

As mentioned earlier, NBs are private companies designated by national authorities to assess conformity with the Regulation of a given type of medical device. In comparison with MDD, the MDR tightens the requirements on NBs. Some of the provisions included in the MDR, however, were introduced when the MDD was the applicable legislation. In 2013, in a response to the breast implants scandal,^35^ the European Commission issued two documents concerning NBs.^36,37^

In the MDR, Articles 35–50 consider NBs. They describe what the designation process and oversight of NBs should look like, referring to the roles of both the national authorities responsible for NBs and the European Commission. The authority responsible for NBs, for example, should review clinical evaluation assessments conducted by NBs to verify their conclusions (Article 45). In addition, Annex VII details requirements that should be met by NBs. The first section of this annex specifies organisational and general requirements that are meant to ensure, among other things, the independence and impartiality of NBs. For example, the MDR prohibits offering consultancy to the manufacturer regarding the device under assessment (Annex VII, 1.2.3 (d)). The subsequent dozen or so pages of Annex VII are devoted to requirements on quality management, personnel and conformity assessment procedures in NBs. Importantly, in the section devoted to conformity assessment activities it is stated that ‘[t]he notified body shall, where relevant, take into consideration available CS [common specifications], guidance and best practice documents and harmonised standards, even if the manufacturer does not claim to be in compliance’. This means that NBs should be aware of and refer to relevant guidelines on medical AI devices. Adherence to this provision may help to prevent common deficiencies in studies on medical AI devices.

In sum, then, the MDR improves oversight and imposes more detailed requirements on NBs that may help to improve the quality and thoroughness of conformity assessments and prevent abuses. This may, in turn, prevent medical devices of unproved safety or performance from entering the market. Jarman et al.^35^ have argued, however, that delegation of the conformity assessment to private entities (i.e. manufacturers and NBs) is a key weakness of the existing regulatory system (also in terms of the safety of devices on the market), and that the recent reforms do not adequately address this. They argue that since the manufacturers are free to choose NBs for the conformity assessment, NBs ‘evolve in a competitive market, compete against each other and adopt market behaviours, which can go against their fundamental public health role’*.*^35^ Jarman et al.^35^ also suggest that the incentives for public bodies to oversee the work of NBs adequately are insufficient, that this gives NBs a relatively weak incentive to conduct appropriate surveillance of manufacturers, and that as a result, the motivation for manufacturers to ensure the safety of their devices is limited.

## Conclusions

The MDR introduces regulatory changes that may contribute to better performance and safety of medical AI devices on the market. The most important modifications appear to centre on new rules for risk classification of the devices, which will result in more devices subjected to a higher degree of scrutiny; more stringent requirements on clinical evaluation, including the requirement for appraisal of the methodological quality and scientific validity of clinical data; new requirements for post-market surveillance, which may help spot early on any new, unexpected side effects and risks of the devices and take corrective actions; and requirements for NBs including for expertise of the personnel and consideration of best practice documents. Other changes, not discussed here, which may also positively impact safety and performance include new harmonised standards (Article 8)^38^ and common specifications (Article 9); the requirement of a ‘person responsible for regulatory compliance’ appointed by the manufacturer within their organisation (Article 15);and provisions regarding devices developed by health institutions for in-house use (Article 5(5)). Furthermore, the MDCG guidance on clinical evaluation of medical device software, and the MEDDEV2.7 guideline on clinical evaluation of medical devices, attend to some of the problems identified in studies on medical AI devices. These documents address the issues mostly in general terms, however, and are not legally binding. Some deficiencies that quite commonly impair studies presenting evidence of the performance of medical AI devices, such as the lack of an adequate external validation of the systems, the lack of randomised clinical trials and multi-site evaluations are not directly addressed in the current guidelines. However, the more stringent regulation of NBs imposed by the MDR, including requirements on adequate expertise of the NB personnel and consideration of best practices, may contribute to a more thorough evaluation of the studies submitted for the conformity assessment and increase the attention paid to the above-mentioned problems. The impact of these provisions depends also on adequate enforcement by the national authorities responsible for NBs.

What steps can be taken to further ensure the safety and performance of medical AI devices on the market? One approach, taken recently by the European Union, is to develop a separate regulation specifically focusing on AI.^39^ The recently proposed Artificial Intelligence Act may help address some of the problems presented by the development of medical AI. For example, Article 10 of the legislative proposal directly addresses the quality of training data and bias.^40^ However, the final version of this regulation, yet to be agreed upon, will take a few years to develop. At this point, a quicker, but not legally binding, option that could help address the problems discussed above might be a guideline issued by the MDCG. This could focus specifically on methodological and reporting standards for studies on medical AI devices and draw on existing guidelines developed by the academic community. The MDCG indeed plans to develop a guideline ‘Artificial Intelligence under MDR/IVDR framework’.^41^ Its content, however, is not yet known.

Importantly, we should keep in mind that regulatory solutions designed to achieve given goals (e.g., safety and quality) may negatively impact other important values or cause some practical problems. For example, a legally binding document outlining detailed rules on the quality of studies on medical AI devices could quickly become outdated due to technological progress and could potentially hamper the development of new, unanticipated technological approaches. Legislators have, therefore, the difficult task of balancing the aims of a given law against any negative impacts of the mechanisms utilised to achieve these aims. Methodological and reporting shortcomings in studies on medical AI devices seem rather common. Since such shortcomings may lead to inaccurate conclusions about safety and performance, and consequently to the presence of unsafe or low-quality medical AI solutions on the market, recent changes in the requirements introduced by the MDR may be justifiable. Yet there may be costs and negative impacts of each of these new requirements, for example, costs for the companies and public authorities (which, in the latter case, are ultimately met by taxpayers), delays in implementation of innovative and beneficial solutions and others, discussion of which is beyond the remit of this article. It is therefore important to keep in mind this broader perspective – including trade-offs between different goals, values and costs – when discussing different regulatory solutions aiming to ensure the safety and quality of medical AI devices.

## Notes

* For the purpose of this article, ‘medical AI device’ means a machine learning-based software intended to be used for a purpose included in the definition of a ‘medical device’ in the Medical Device Regulation (Article 2). This definition overlaps with the definition of ‘AL/ML-based software as a medical device’ used by the FDA, however, there are differences in the meaning of the term ‘medical device’ in the MDR and U.S. legislation.
